# Characteristics of *Pseudomonas aeruginosa* infection in intensive care unit before (2007–2010) and after (2011–2014) the beginning of an antimicrobial stewardship program

**DOI:** 10.1017/ash.2024.53

**Published:** 2024-04-29

**Authors:** Alessio Strazzulla, Vladimir Adrien, Segla Robert Houngnandan, Sandra Devatine, Ouerdia Bahmed, Sarra Abroug, Sarra Hamrouni, Mehran Monchi, Sylvain Diamantis

**Affiliations:** 1 Internal and General Medicine Unit, Groupe Hospitalier Sud Ile de France, Melun, France; 2 Infectious Diseases Unit, Groupe Hospitalier Sud Ile de France, Melun, France; 3 Department of Infectious and Tropical Diseases, Avicenne Hospital, AP-HP, Université Sorbonne Paris Nord, Bobigny, France; 4 Intensive Care Unit, Groupe Hospitalier Sud Ile de France, Melun, France; 5 EA 7380 Dynamic, Université Paris Est Créteil, EnvA, USC ANSES, Créteil, France

## Abstract

**Objectives:**

To investigate the factors associated with *Pseudomonas aeruginosa* isolates in intensive care unit (ICU) before and after an antimicrobial stewardship program.

**Materials:**

Monocentric retrospective cohort study. Patients admitted to the ICU in 2007–2014 were included. Characteristics of *P. aeruginosa* patients were compared to overall ICU population. Clinical and microbiological characteristics of *P. aeruginosa* patients before (2007–2010) and after (2011–2014) the beginning of the AMP were compared.

**Results:**

Overall, 5,263 patients were admitted to the ICU, 274/5,263 (5%) had a *P. aeruginosa* isolate during their staying. In 2011–2014, the percentage *P. aeruginosa* isolates reduced (7% vs 4%, *P* ≤ .0001). Patients with *P. aeruginosa* had higher rates of in-hospital death (43% *vs* 20%, *P* < .0001) than overall ICU population. In 2011–2014, rates of multidrug-resistant (11% *vs* 2%, *P* = .0020), fluoroquinolone-resistant (35% vs 12%, *P* < .0001), and ceftazidime-resistant (23% vs 8%, *P* = .0009) *P. aeruginosa* reduced. Treatments by fluoroquinolones (36% vs 4%, *P* ≤ .0001), carbapenems (27% vs 9%, *P* = .0002), and third-generation cephalosporins (49% vs 12*%*, *P* ≤ .0001) before *P. aeruginosa* isolation reduced while piperacillin (0% vs 13%, *P* < .0001) and trimethoprim-sulfamethoxazole (8% vs 26%, *P* = .0023) increased. Endotracheal intubation reduced in 2011–2014 (61% vs 35%, *P* < .0001). Fluoroquinolone-resistance was higher in patients who received endotracheal intubation (29% vs 17%, *P* = .0197). Previous treatment by fluoroquinolones (OR = 2.94, *P* = .0020) and study period (2007–2010) (OR = 2.07, *P* = .0462) were the factors associated with fluoroquinolone-resistance at the multivariate analysis.

**Conclusions:**

Antibiotic susceptibility in *P. aeruginosa* isolates was restored after the reduction of endotracheal intubation, fluoroquinolones, carbapenems, and third-generation cephalosporins and the increased use of molecules with a low ecological footprint, as piperacillin and trimethoprim-sulfamethoxazole.

## Introduction

*Pseudomonas aeruginosa* can be responsible of life-threatening diseases, as a consequence of urinary, bone, respiratory, abdominal, and disseminated infections.^1^ It colonizes human body, being part of the human microbiota (especially in the respiratory tract), and it can also be acquired from exogenous sources.^2^ Patients hospitalized in intensive care unit (ICU) are at risk of contamination. Potential exogenous sources are tap-water and fomites while patient-to-patient transmission is possible but it can be limited by standard precautions.^3^ Antibiotic pressure is the most relevant factor for *P. aeruginosa* acquisition in ICU.^4^

At each antibiotic treatment, germs of the human microbiota are exposed to sub-lethal levels of antibiotics. This event enhances the selection of antibiotic resistance genes, which often are host in transferable plasmids.^5^ Otherwise, the antibiotic pressure can trigger chromosomal mutations and transfer of resistance determinants, as is typical for *P. aeruginosa.* ^6^ Because of the risk of antibiotic resistance, the use of broad-spectrum molecules is currently discouraged as antibiotic prophylaxis and treatment.^7^ The benefits in terms of antimicrobial susceptibility resulting from the reduced consumption of broad-spectrum molecules in ICU was largely demonstrated for gram-negative bacilli.^8^ However, the results of antimicrobial stewardship programs (ASPs) in ICU concerning *P. aeruginosa* are often deceiving with no significant reduction of antimicrobial resistance rates.^9–11^

At the end of 2010, an ASP started at the ICU of the Melun General Hospital, a 350-bed tertiary care hospital in the *Ile-de-France* region in France, its ICU accounting for a total of 24 beds. The main objective of the ASP was to restrain the consumption of broad-spectrum molecules (carbapenems, fluoroquinolones (FLQ), and third-generation cephalosporins (3-GC)). This objective was fully achieved. Indeed, a reduction of 50%–85% in the consumption of carbapenems, FLQ, and 3-GC was observed in the following 4-year period (from 2011 to 2014) when compared to the previous one (from 2007 to 2010). Contemporarily, a reduction of AmpC hyperproducing group 3 *Enterobacteriaceae*, FLQ resistant, and ceftazidime resistant *P. aeruginosa* was observed.^12^

This study pursues the previous one through a *P. aeruginosa* targeted analysis. It investigates the factors associated with isolation of *P. aeruginosa* from ICU patients before and after the beginning of the ASP, with a special focus on the risk of antibiotic-resistance and the benefits produced by the ASP in terms of antibiotic susceptibility recovering.

## Materials and methods

A monocentric retrospective cohort study was conducted at Melun General Hospital, a 350 tertiary bed hospital in Melun (France). Patients were hospitalized in ICU, accounting for 24 beds. All adult patients admitted to the ICU presenting *P. aeruginosa* isolates during their hospitalization in ICU from January the 1^st^, 2007 to December 31^st^, 2014 were included. Two timeframes were analyzed: (1) before the beginning of the ASP (2007–2010); (2) after the beginning of the ASP (2011–2014).

The previous study by *Abbara et al* already evaluated the susceptibility of all *P. aeruginosa* isolated from any site in patients hospitalized in ICU during the same study period. It focused on resistance to single molecules and did not explored the factors associated with *P. aeruginosa* isolation.^12^ For this study, we revised all ICU patient’s files and selected patients with *P. aeruginosa* isolation from any site during ICU stay and up to 7 days after ICU discharge to explore the possible late impact of ICU stay on *P. aeruginosa* selection. We included all *P. aeruginosa* isolates, both infections and colonizations, while in the previous study only isolates of clinical significance were included. We also added analysis about multidrug resistant (MDR) *P. aeruginosa*. The characteristics of patients with *P. aeruginosa* isolation were compared to ICU population and an analysis before/after intervention was performed.

The study was conducted in accordance with Declaration of Helsinki and national and institutional standards.^13^

Data were obtained through the revision of patients’ files which were collected in software used in daily clinical practice (Sillage v17 and CGM Lab channel 1.20.33686). Microbiological identifications and susceptibility tests were performed according to recommendations of the European committee on antimicrobial susceptibility testing.^14^ The following outcomes were considered: (1) acquisition MDR bacteria; (2) length of ICU stay; (3) length of hospital stay; (4) in-ICU death; and (5) in-hospital death.

Fisher’s exact test (qualitative variables) and Student’s t-test (quantitative variables) were applied for the univariate analysis. At first, characteristics of *P. aeruginosa* patients were compared to overall ICU population. Then, clinical and microbiological characteristics of *P. aeruginosa* patients before (2007–2010) and after (2011–2014) the beginning of the AMP were compared. Analysis according to the origin of the infection (community acquired *vs* hospital acquired) was also performed. Logistic regression analysis was performed for multivariate analysis. For the multivariate analysis of risk factors of FLQ resistance the parameters included in the analysis were chosen according to univariate analysis results (*P* ≤ .0001). For the analysis of the risk of MDR *P. aeruginosa* only the exposition to any class of antibiotics was considered. Statistical significance was set at *P* < .050.

## Results

Overall, 5,263 patients were admitted to the ICU during the study period (2007–2014), 274/5263 (5%) having at least a *P. aeruginosa* isolate during hospitalization in ICU and up to 7 days after ICU discharge. The percentage of patients with *P. aeruginosa* isolates reduced significantly before and after the ASP (7% in 2007–2010 vs 4% in 2011–2014, *P* ≤ .0001). Patients with *P. aeruginosa* had longer hospital stays and higher rates of in-hospital and in-ICU death (*P* < .0001) than overall ICU population. They received endotracheal intubation more frequently than patients without *P. aeruginosa* isolates (*P* < .0001). Table 1 resumes characteristics of the study population.

**Table 1.** Characteristics of the population

Characteristics	*Pseudomonas aeruginosa*		
	No	Yes	*P* value
	*n* = 4,989	*n* = 274	
Median age, years [min–max]	65 [51–78]	73 [58–81]	<.0001
Male gender, *n* [%]	2,929 [57]	180 [64]	<.0001
SAPS-II at ICU admission, mean [%]	39.7 [25.4]	48.8 [18.8]	.59
Fluid resuscitation, *n* [%]	1004 [20]	63 [23]	.14
Renal replacement therapy, *n* [%]	390 [8]	44 [16]	<.0001
Vasopressors, *n* [%]	1590 [32]	172 [63]	<.0001
Central venal catheter, *n* [%]	2169 [43]	215 [78]	<.0001
Endotracheal intubation, *n* [%]	887 [18]	130 [47]	<.0001
MV with PEEP ≤ 6 cmH_2_O and FiO_2_ ≤ 60%, *n* [%]	1293 [26]	147 [54]	<.0001
MV with PEEP ≥ 6 cmH_2_O and FiO_2_ ≥ 60%, *n* [%]	1202 [24]	140 [51]	<.0001
MV with PEEP ≥ 6 and FiO_2_ ≥ 60% cmH_2_O and ventral decubitus, *n* [%]	80 [1.6]	10 [4]	.0182
ICU length of stay, days, mean [SD]	3.2 [6.1]	9.1 [16.9]	<.0001
Hospital length of stay, days, mean [SD]	15.2 [19.1]	41.3 [40.4]	<.0001
In-ICU death, *n* [%]	840 [17]	102 [37]	<.0001
In-hospital death, *n* [%]	995 [20]	118 [43]	<.0001

Note. CI, confidence interval; ICU, intensive care unit; FiO2, fraction of inspired oxygen; MV, mechanical ventilation; PEEP, positive end-expiratory pressure; RR, related ratio; SAPS-II, simplified acute physiology score-II; SD, standard deviation.

In 2011–2014, rates of multidrug-resistant, fluoroquinolone-resistant, and ceftazidime-resistant *P. aeruginosa* reduced significantly (*P* = .0020, *P* < .0001 and *P* = .0009, respectively). Rates of antibiotic treatments by FLQ, carbapenems, and 3-GC before *P. aeruginosa* isolation reduced significantly in 2011–2014 (*P* ≤ .0002). Simultaneously, use of piperacillin (without tazobactam) and trimethoprim-sulfamethoxazole (TMP-SMX) increased (*P* < .0001 and *P* = .0023, respectively). *P. aeruginosa* patients received endotracheal intubation and mechanical ventilation less frequently in 2011–2014 than 2007–2010. Also, the length of hospital and ICU stay decreased during the same period (*P* < 0.0001 and *P* = 0.08, respectively). Table 2 shows characteristics of the population with *P. aeruginosa* isolates.

**Table 2.** Characteristics of patients with *Pseudomonas aeruginosa* isolates

Characteristics	*Pseudomonas aeruginosa*			
	Overall	2007–2010	2011–2014	*P* value
	*n* = 274	*n* = 132	*n* = 142	
**Patients’ biological parameters and medical history**				
Median age, years [min–max]	72.5 [58–81]	75 [60.5–82]	70 [59–78.5]	.02
Male gender, *n* [%]	177 [65]	77 [57]	100 [70]	.02
Chronic obstructive pulmonary disease, *n* [%]	89 [32]	49 [37]	40 [28]	.07
Alcohol addiction, *n* [%]	43 [16]	26 [20]	17 [12]	.06
Diabetes, *n* [%]	59 [22]	32 [24]	27 [19]	.18
Immunodepression, *n* [%]	24 [9]	4 [3]	20 [14]	.0007
Traumatic brain injury, *n* [%]	16 [5.8]	9 [7]	7 [5]	.34
Wounds, *n* [%]	82 [30]	36 [27]	46 [32]	.21
**Hospital stay in ICU**				
SAPS-II at ICU admission, mean [SD]	48.7 [19.8]	48.6 [20.7]	48.8 [18.9]	.93
Fluid resuscitation, *n* [%]	63 [23]	10 [8]	53 [37]	<.0001
Renal replacement therapy, *n* [%]	44 [16]	7 [5]	37 [26]	<.0001
Vasopressors, *n* [%]	172 [63]	94 [71]	78 [55]	.0042
Central venal catheter, *n* [%]	215 [78]	118 [89]	97 [68]	<.0001
Continued noninvasive ventilation, *n* [%]	10 [4]	2 [2]	8 [6]	.07
Endotracheal intubation, *n* [%]	130 [47]	80 [61]	50 [35]	<.0001
MV with PEEP ≤ 6 cmH_2_O and FiO_2_ ≤ 60%, *n* [%]	147 [54]	96 [73]	51 [36]	<.0001
MV with PEEP ≥ 6 cmH_2_O and FiO_2_ ≥ 60%, *n* [%]	140 [51]	68 [52]	72 [51]	.49
MV with PEEP ≥ 6 cmH_2_O and FiO_2_ ≥ 60% and ventral decubitus, *n* [%]	10 [4]	1 [1]	9 [6.3]	.0132
Length of invasive mechanical ventilation, days, mean [SD]	22.5 [24.3]	29.7 [29.8]	15.9 [14.8]	<.0001
Length of intratracheal intubation, days, mean [SD]	14.5 [13.2]	16.6 [13.3]	12.5 [12.8]	.0168
**Previous antibiotic treatments**				
Antibiotic treatment before *Pseudomonas aeruginosa, n* [%]	247 [90]	122 [92]	125 [88]	.22
Amoxicillin/clavulanic acid before *Pseudomonas aeruginosa, n* [%]	68 [25]	38 [29]	30 [21]	.14
Piperacillin before *Pseudomonas aeruginosa, n* [%]	18 [7]	0 [0]	18 [13]	<.0001
Piperacillin/tazobactam before *Pseudomonas aeruginosa, n* [%]	104 [38]	44 [33]	60 [42]	.13
Ceftriaxone/cefotaxime before *Pseudomonas aeruginosa, n* [%]	53 [19]	39 [30]	14 [10]	<.0001
Ceftazidime before *Pseudomonas aeruginosa, n* [%]	29 [11]	26 [20]	3 [2]	<.0001
Cefepime before *Pseudomonas aeruginosa, n* [%]	3 [1]	1 [1]	1 [1]	NA
Carbapenem before *Pseudomonas aeruginosa, n* [%]	48 [18]	35 [27]	13 [9]	.0002
Aminoglycoside before *Pseudomonas aeruginosa, n* [%]	123 [45]	57 [51]	56 [39]	.06
Glycopeptide before *Pseudomonas aeruginosa, n* [%]	50 [18]	40 [30]	10 [7]	<.0001
Fluoroquinolone before *Pseudomonas aeruginosa, n* [%]	54 [20]	48 [36]	6 [4]	<.0001
Macrolide/lincosamide before *Pseudomonas aeruginosa, n* [%]	38 [14]	9 [7]	29 [20]	.0011
Trimethoprim/sulfamethoxazole before *Pseudomonas aeruginosa, n* [%]	47 [17]	10 [8]	37 [26]	.0023
Tetracyclines before *Pseudomonas aeruginosa, n* [%]	2 [1]	1 [1]	1 [1]	NA
Colistin before *Pseudomonas aeruginosa, n* [%]	17 [6]	15 [11]	2 [1]	.0006
Other molecules* before *Pseudomonas aeruginosa, n* [%]	26 [9]	10 [8]	16 [11]	.30
**Risk factors of MDR** ***Pseudomonas aeruginosa***				
Antibiotic therapy <6 mo, *n* [%]	93 [34]	45 [34]	48 [34]	.53
Hospitalization <6 mo, *n* [%]	123 [45]	63 [48]	60 [42]	.22
Other infection than *Pseudomonas aeruginosa*, *n* [%]	188 [69]	96 [73]	92 [65]	.1
Previous *Pseudomonas aeruginosa* infection, *n* [%]	22 [8]	9 [7]	13 [9]	.31
**Characteristics of** ***Pseudomonas aeruginosa*** **isolates**				
Δ date of ICU admission - date of *Pseudomonas aeruginosa* isolation, mean [SD]	10 [30]	13 [35]	8 [23]	.1618
*Pseudomonas aeruginosa* isolation > day 7 of stay in ICU, *n* [%]	84 [31]	39 [30]	45 [42]	.67
*Pseudomonas aeruginosa* colonization, *n* [%]	95 [35%]	39 [30%]	56 [39%]	.055
*Pseudomonas aeruginosa* infection, *n* [%]	179 [65%]	93 [70%]	86 [61%]	.055
*Pseudomonas aeruginosa* in respiratory tract, *n* [%]	120 [44]	58 [44]	62 [44]	.45
*Pseudomonas aeruginosa* in bloodstream, *n* [%]	13 [5]	6 [5]	7 [5]	.86
*Pseudomonas aeruginosa* in urine, *n* [%]	36 [13]	16 [12]	20 [14]	.53
*Pseudomonas aeruginosa* in other sites**, *n* [%]	24 [9]	12 [9]	12 [9]	.56
*Pseudomonas aeruginosa* VAP*, n* [%]	92 [34]	44 [33]	48 [34]	.52
*Pseudomonas aeruginosa* sepsis*, n* [%]	24 [10]	12 [9]	12 [8.5]	.51
Ceftazidime resistant *Pseudomonas aeruginosa*, *n* [%]	41 [15]	30 [23]	11 [8]	.0009
Ceftazidime resistant *Pseudomonas aeruginosa* > day 7 of stay, *n* [%]	8 [3]	7 [5]	1 [1]	.93
Imipenem resistant *Pseudomonas aeruginosa*, *n* [%]	37 [14]	21 [16]	16 [11]	.26
Imipenem resistant *Pseudomonas aeruginosa* > day 7 of stay, *n* [%]	9 [3]	5 [4]	4 [3]	.28
Fluoroquinolone resistant *Pseudomonas aeruginosa*, *n* [%]	63 [23]	46 [35]	17 [12]	<.0001
Fluoroquinolone resistant *Pseudomonas aeruginosa* > day 7 of stay, *n* [%]	5 [14]	10 [8]	4 [3]	.91
MDR *Pseudomonas aeruginosa*, *n* [%]	18 [7]	15 [11]	3 [2]	.0020
MDR *Pseudomonas aeruginosa* > day 7 of stay, *n* [%]	4 [1]	3 [2]	1 [1]	.61
**Outcomes**				
Overall MDR bacteria, *n* [%]	74 [27]	43 [33]	31 [22]	.03
Hospital acquired MDR bacteria, *n* [%]	37 [14]	22 [17]	15 [11]	.1
ICU length of stay (days), mean [SD]	9.1 [16.9]	11 [20.3]	7.4 [12.7]	.08
Hospital length of stay (days), mean [SD]	41.3 [40.4]	53 [50.7]	30.3 [22.9]	<.0001
In-ICU death, *n* [%]	102 [37]	47 [36]	55 [39]	.34
In-hospital death, *n* [%]	118 [43]	53 [40]	65 [46]	.21

Note. *including: linezolid, fosfomycin, daptomycin, rifampicin; **including: purulent lesions, cutaneous biopsies, vascular catheter, bone biopsies, coprocultures and peritoneal fluids; CI, confidence interval; ICU, intensive care unit; FiO_2_, fraction of inspired oxygen; MDR, multidrug resistant; MV, mechanical ventilation; NA, not applicable; PEEP, positive end-expiratory pressure; RR, related ratio; SAPS-II, simplified acute physiology score-II; SD, standard deviation; VAP, ventilator associated pneumonia.

Patients with hospital acquired *P. aeruginosa* had higher rates of endotracheal intubation (*P* < .0001), central venous catheter (*P* = .0316), and previous FLQ treatment (*P* = .0059) than patients with community acquired *P. aeruginosa*. Sepsis was more frequent (*P* = .0063) among patient with community acquired *P. aeruginosa* than patients with hospital acquired *P. aeruginosa* (Table 3).

**Table 3.** Characteristics of patients with *Pseudomonas aeruginosa* isolates

Characteristics	*Pseudomonas aeruginosa*			
	Overall	Nosocomial	Community acquired	*P* value
	*n* = 274	*n* = 213	n = 61	
**Patients’ biological parameters and medical history**				
Median age, years [min–max]	72.5 [58–81]	72 [15–92]	74 [33–91]	.02
Male gender, *n* [%]	180 [64]	137 [65]	40 [66]	.49
Chronic obstructive pulmonary disease, *n* [%]	89 [32]	69 [33]	20 [33]	.53
Alcohol addiction, *n* [%]	43 [16]	36 [17]	7 [12]	.21
Diabetes, *n* [%]	59 [22]	50 [24]	9 [15]	.10
Traumatic brain injury, *n* [%]	16 [5.8]	14 [7]	2 [13]	.26
Wounds, *n* [%]	82 [30]	64 [30]	18 [29]	.53
**Hospital stay in ICU**				
SAPS-II at ICU admission, mean [SD]	48.7 [19.8]	47.7 [18.6]	51.9 [23.1]	.10
Fluid resuscitation, *n* [%]	63 [23]	42 [20]	21 [34]	.0146
Renal replacement therapy, *n* [%]	44 [16]	33 [16]	11 [18]	.38
Vasopressors, *n* [%]	172 [63]	138 [65]	34 [56]	.13
Central venal catheter, *n* [%]	215 [78]	173 [81]	42 [68]	.0316
Continued non-invasive ventilation, *n* [%]	10 [4]	8 [4]	2 [3]	.61
Endotracheal intubation, *n* [%]	130 [47]	117 [55]	13 [21]	<.0001
MV with PEEP ≤ 6 cmH_2_O and FiO_2_ ≤ 60%, *n* [%]	147 [54]	125 [59]	22 [36]	.0014
MV with PEEP ≥ 6 cmH_2_O and FiO_2_ ≥ 60%, *n* [%]	140 [51]	11 [52]	29 [48]	.31
MV with PEEP ≥ 6 cmH_2_O and FiO_2_ ≥ 60% and ventral decubitus, *n* [%]	10 [4]	10 [5]	0 [0.0]	.07
Length of invasive mechanical ventilation, days, mean [SD]	22.5 [24.3]	25.4 [24.1]	12.3 [21.8]	.0002
Length of intratracheal intubation, days, mean [SD]	14.5 [13.2]	16.2 [12.2]	8.1 [14.3]	<.0001
**Previous antibiotic treatments**				
Antibiotic treatment before *Pseudomonas aeruginosa, n* [%]	247 [90]	196 [92]	51 [84]	.04
Amoxicillin/clavulanic acid before *Pseudomonas aeruginosa, n* [%]	68 [25]	57 [27]	11 [18]	.10
Piperacillin before *Pseudomonas aeruginosa, n* [%]	18 [7]	16 [8]	2 [3]	.19
Piperacillin/tazobactam before *Pseudomonas aeruginosa, n* [%]	104 [38]	80 [38]	24 [39]	.45
Ceftriaxone/cefotaxime before *Pseudomonas aeruginosa, n* [%]	53 [19]	45 [21]	8 [13]	.11
Ceftazidime before *Pseudomonas aeruginosa, n* [%]	29 [11]	25 [12]	4 [7]	.18
Cefepime before *Pseudomonas aeruginosa, n* [%]	3 [1]	3 [1]	0 [0]	.46
Carbapenem before *Pseudomonas aeruginosa, n* [%]	48 [18]	41 [19]	7 [11]	.11
Aminoglycoside before *Pseudomonas aeruginosa, n* [%]	123 [45]	98 [46]	25 [39]	.28
Glycopeptide before *Pseudomonas aeruginosa, n* [%]	50 [18]	39 [18]	11 [18]	.55
Fluoroquinolone before *Pseudomonas aeruginosa, n* [%]	54 [20]	49 [23]	5 [8]	.0059
Macrolide/lincosamide before *Pseudomonas aeruginosa, n* [%]	38 [14]	30 [14]	8 [13]	.52
Trimethoprim/sulfamethoxazole before *Pseudomonas aeruginosa, n* [%]	47 [17]	39 [18]	8 [13]	.23
Tetracyclines before *Pseudomonas aeruginosa, n* [%]	2 [1]	2 [1]	0 [0]	.60
Colistin before *Pseudomonas aeruginosa, n* [%]	17 [6]	14 [7]	3 [5]	.77
Other molecules* before *Pseudomonas aeruginosa, n* [%]	26 [9]	23 [11]	3 [5]	.12
**Risk factors of MDR** ***Pseudomonas aeruginosa***				
Antibiotic therapy < 6 mo, *n* [%]	93 [34]	69 [32]	24 [39]	.19
Hospitalization < 6 mo, *n* [%]	123 [45]	86 [40]	37 [61]	.0040
Other infection than *Pseudomonas aeruginosa*, *n* [%]	188 [69]	163 [76]	25 [40]	<.0001
Previous *Pseudomonas aeruginosa* infection, *n* [%]	22 [8]	13 [6]	9 [15]	.0324
**Characteristics of** ***Pseudomonas aeruginosa*** **isolates**				
*Pseudomonas aeruginosa* colonization, *n* [%]	95 [35%]	75 [35%]	20 [33%]	.42
*Pseudomonas aeruginosa* infection, *n* [%]	179 [65%]	138 [64%]	41 [67%]	.42
*Pseudomonas aeruginosa* in respiratory tract, *n* [%]	146 [53]	99 [47]	21 [35]	.05
*Pseudomonas aeruginosa* in urine, *n* [%]	36 [13]	27 [13]	9 [15]	.40
*Pseudomonas aeruginosa* in other sites**, *n* [%]	24 [9]	13 [6]	11 [18]	.0062
*Pseudomonas aeruginosa* sepsis*, n* [%]	24 [10]	13 [6]	11 [18]	.0063
Ceftazidime resistant *Pseudomonas aeruginosa*, *n* [%]	41 [15]	32 [15]	9 [15]	.57
Imipenem resistant *Pseudomonas aeruginosa*, *n* [%]	37 [14]	33 [15]	4 [7]	.0497
Fluoroquinolone resistant *Pseudomonas aeruginosa*, *n* [%]	63 [23]	54 [25]	9 [15]	.06
MDR *Pseudomonas aeruginosa*, *n* [%]	18 [7]	16 [8]	2 [3]	.19
**Outcomes**				
Overall MDR bacteria, *n* [%]	74 [27]	61 [29]	13 [22]	.18
ICU length of stay (days), mean [SD]	9.1 [16.9]	10.4 [18.0]	4.4 [10.7]	.032
Hospital length of stay (days), mean [SD]	41.3 [40.4]	46.3 [40.3]	23.6 [35.6]	.0001
In-ICU death, *n* [%]	102 [37]	76 [36]	26 [42]	.20
In-hospital death, *n* [%]	118 [43]	89 [42]	29 [48]	.25

Note. *including linezolid, fosfomycin, daptomycin, rifampicin; **including purulent lesions, cutaneous biopsies, vascular catheter, bone biopsies, coprocultures and peritoneal fluids; CI, confidence interval; ICU, intensive care unit; FiO2, fraction of inspired oxygen; MDR, multidrug resistant; MV, mechanical ventilation; NA, not applicable; PEEP, positive end-expiratory pressure; RR, related ratio; SAPS-II, simplified acute physiology score-II; SD, standard deviation; VAP, ventilator associated pneumonia.

The univariate analysis showed that patients who received endotracheal intubation had higher rates of fluoroquinolone resistant *P. aeruginosa* isolation (*P* = .0197; Table 4). At the multivariate analysis, the factors associated with fluoroquinolone-resistance were study period (2007–2010) and previous treatment by fluoroquinolones (*P* = 0.0020 and *P* = 0.0462, respectively), as shown in Table 5. No previous use of any class of antibiotics was associated with the risk of MDR *P. aeruginosa* (Table 6).

**Table 4.** Resistance to fluoroquinolones among *Pseudomonas aeruginosa* isolates from patients receiving endotracheal intubation

Characteristics	Endotracheal intubation		
	Yes	No	*P* value
**Overall (** ***n*** **= 274)**			
Fluoroquinolone resistant isolates, *n* [%]	38 [29]	25 [17]	.0197
Fluoroquinolone resistant isolates in patients treated by fluoroquinolones, *n* [%]	18 [46]	5 [20]	.0273
**Respiratory samples (** ***n*** **= 146)**			
Fluoroquinolone resistant isolates, *n* [%]	26 [35]	14 [24]	.0259
Fluoroquinolone resistant isolates in patients treated by fluoroquinolones, *n* [%]	12 [54]	3 [21]	.0495

**Table 5.** Multivariate analysis of factors associated with fluoroquinolone resistance in *Pseudomonas aeruginosa* isolates

Characteristics	OR (95% CI)	*P* value
Length of endotracheal intubation, days	1.39 (0.6–3.2)	.44
Length of invasive mechanical ventilation	0.60 (0.3–1.4)	.23
Continued non-invasive ventilation	1.30 (0.2–6.7)	.76
Previous fluoroquinolone treatment	2.07 (1.0–4.2)	.0462
Study period (2007–2010 vs 2011–2014)	2.94 (1.5–5.8)	.0020

**Table 6.** Multivariate analysis of factors associated with presence of multi drug resistant *Pseudomonas aeruginosa* isolates

Characteristics	OR (95% CI)	*P* value
Previous aminoglycoside	1.82 (0.6–5.1)	.26
Previous cephalosporin	0.78 (0.2–2.5)	.68
Previous carbapenem	1.17 (0.3–3.9)	.80
Previous tetracycline	0.0001 (NA)	.98
Previous fluoroquinolone	2.13 (0.7–6.9)	.20
Previous macrolide	1.63 (0.4–6.2)	.47
Previous penicillin	0.40 (0.1–1.2)	.09
Previous Trimethoprim/sulfamethoxazole	0.31 (0.03–2.4)	.27

Note. NA, not applicable.

## Discussion

This study showed that the frequency of *P. aeruginosa* infection in ICU reduced after the beginning of an ASP, principally based on saving of broad-spectrum antibiotics. The antimicrobial susceptibility of *P. aeruginosa* recovered after the reduction of 3-GC and fluoroquinolone consumption and the increased prescription of alternative “old” molecules, such as piperacillin and trimethoprim-sulfamethoxazole. Endotracheal intubation was associated with FLQ resistance.

Although ASP are strongly recommended, reducing antibiotic consumption in ICU is extremely difficult because patients’ uncertain diagnosis and compromised hemodynamic state push prescribers keeping long-course broad-spectrum antibiotic treatments. A consequence of this attitude is an increased risk of *P. aeruginosa* infection which occurs almost exclusively in patients who received a previous antibiotic treatment.^15^ In fact, broad-spectrum antibiotics interact with the environment and facilitate *P. aeruginosa* acquisition.^4^ Our ASP succeeded in reducing both *P. aeruginosa* antibiotic resistance and infection incidence. One of the key of this success was the increased use of molecules with low ecological footprint to treat infections others than *P. aeruginosa*.

The use of FLQ as empirical treatment for *P. aeruginosa* is inadvisable because of the risk of treatment failure and the increased mortality due to the development of antibiotic resistance.^16^ Indeed, *P. aeruginosa* has many mechanisms of resistance, frequently based on *GyrA*, *ParC,* and *MexR* enzymes.^17^ In ICU setting, an important virulence and resistance factor is biofilm which contributes reducing susceptibility to antimicrobial molecules and host-immune factors.^18^ This effect disappears when bacteria are deprived of the capacity of producing the extracellular matrix made by polysaccharides, proteins, and metabolites.^19^ As a consequence, biofilm reduces antibiotic efficacy by several mechanisms: reduction of antibiotic penetration, microenvironment modifications, and increased inflammatory response.^20^ FLQ activity is largely limited by biofilm production.^21^ In ICU, the main sources of biofilm producing *P. aeruginosa* are invasive devices.^4^ Moreover, endotracheal intubation is the most relevant determinant of *P. aeruginosa* acquisition and ventilator-associated pneumonia (VAP).^22^ The restriction of endotracheal intubation was adopted in our ICU to reduce respiratory infection rate. It was obtained through new standard of care, such as protocol-based sedation, favoring noninvasive ventilation over invasive ventilation whenever possible, and improved ventilation weaning process in mechanical ventilation. No change in devices and patient admission policy was adopted during the study period. Results of this study are in line with other studies which showed that restriction of endotracheal intubation was associated with reduction of mortality and MDR bacterial infection.^23,24^ This study showed that previous FLQ treatment was associated with FLQ resistance in *P. aeruginosa* strains afterward isolated. It also showed that patients who received endotracheal intubation had higher rates of FLQ resistance. We can hypothesize that the reduction of endotracheal intubation observed from 2011 to 2014 could have contributed in reducing rates of FLQ resistant *P. aeruginosa*. This study advocates against the use of FLQ in intubated patients because of the increased risk of FLQ resistance in *P. aeruginosa* strains.

FLQ are frequently prescribed for atypical pneumonia and intracellular bacterial infections but their collateral damages in term of selection of MDR bacterial impose their limitation as empirical antibiotic treatment. For this reason, our ASP suggested macrolides as alternative molecules.^12^ Indeed, macrolides can be preferred to FLQ in many situations. At first, macrolides are not inferior to FLQ for the treatment of *Legionella* pneumonia.^25^ Second, the treatment of severe community acquired pneumonia with beta-lactam plus macrolides resulted more effective than treatment with FLQ alone in reducing mortality and length of hospitalization in ICU.^26^ Third, because of their immunomodulatory effects, macrolides are an interesting alternative for the treatment of low respiratory tract infections in patients affected by chronic respiratory diseases.^27^ In this study, macrolides contributed to reduce FLQ prescriptions.

Piperacillin is a broad-spectrum beta-lactam. It is active against gram-positive bacteria and it shows high activity against gram-negative bacilli, both aerobic and anaerobic (*Klebsiella pneumoniae*, *Serratia marcescens*, and *P. aeruginosa*).^28^ It is hydrolyzed by beta-lactamases (as TEM-1) and, therefore, it is almost always administrated in association with tazobactam, a beta-lactamase inhibitor which successfully restores the activity of piperacillin against many beta-lactamases.^29^ However, *P. aeruginosa* may rapidly develop resistance to tazobactam by the production of extended spectrum beta-lactamases and AmpC beta-lactamases.^30^ The use of piperacillin “alone” without the adding of tazobactam for documented infection caused by gram-negative bacteria was adopted in our ICU with the rationale of sparing tazobactam and, therefore, reducing the antimicrobial selective pressure on targeted pathogens, bacteria of the human microbiota and invasive device’s contaminants. Results of this study suggest that this strategy could have contributed in reducing rates of MDR and ceftazidime resistant *P. aeruginosa* strains. Further studies are needed to confirm this hypothesis.

TMP-SMX represents an alternative to FLQ and beta-lactams for the treatment of infection by gram-negative (*Enterobacteriaceae*) and gram-positive (*Staphylococcus aureus)* bacteria, although it is not active against *P. aeruginosa.* In France, it is currently the first choice for treatment of documented urinary infection according to French national recommendations.^31^ In our establishment, TMP-SFX is successfully used for the treatment of VAP by bacteria other than *P. aeruginosa.* ^32^ According to our ASP, TMP-SMX was preferred to FLQ and beta-lactams whenever the antimicrobial susceptibility test confirmed the sensibility to TMP-SMX. Aim of this choice was to reduce antibiotic “collateral damages” and in particular the selection of MDR bacteria. The reduction of *P. aeruginosa’s* resistance rates to FLQ and beta-lactams was likely influenced by the reduced consumption of broad-spectrum molecules (FLQ and 3-GC) and their replacement by molecules with a lower ecological footprint, such as TMP-SMX and piperacillin.

Results of this study were limited by its retrospective design. Indeed, a loss of data was expected and direct comparison between molecules were not possible. Also, a longer period analysis was necessary to confirm the positive results of the ASP. Because of the study design, the number of variables was limited and many factors potentially associated with *P. aeruginosa* isolation were not investigated. Notwithstanding, results of this study are encouraging and justify the pursue of exploration by further studies. In particular, factors associated with *P. aeruginosa* isolates occurring in patients hospitalized in non-ICU units needs to be explored. Also, risk factors of *P. aeruginosa* VAP need to be investigated. A direct comparison between different molecules necessitates to be performed.

## Conclusions

The antibiotic stewardship program implemented in our institution achieved in reducing rates of antibiotic resistance in *P. aeruginosa* isolates obtained from ICU patients. Among the factors investigated by this study, the decreasing consumption of 3-GC and FLQ and the increased use of TMP-SMX and piperacillin contributed in achieving this result. Also, the decreasing use of endotracheal intubation was observed and likely participate in reducing rates of *P. aeruginosa* isolation. Further studies are needed to verify the effectiveness of this strategy in other settings.
